# Does prior knowledge affect interaction dynamics and learning achievement in digital problem-based learning? A pilot study

**DOI:** 10.3205/zma001651

**Published:** 2023-11-15

**Authors:** Martin Möser, Rico Hermkes, Natalie Filmann, Seon-Yee Harsch, Stefan Rüttermann, Susanne Gerhard-Szép

**Affiliations:** 1 Goethe University Frankfurt, Department of Operative Dentistry, Frankfurt/Main, Germany; 2 Goethe University Frankfurt, Department of Business Ethics and Business Education, Frankfurt/Main, Germany; 3 Goethe University Frankfurt, Department of Biostatistics and Mathematical Modelling, Frankfurt/Main, Germany

**Keywords:** problem-based learning, PBL, video-study, digital, interaction, prior knowledge, learning achievement

## Abstract

**Objective:**

Previous research on problem-based learning (PBL) describes that videotaped observations develop meaningful insights into cognitive processes in tutorial groups. Analysis regarding the amount of prior knowledge on learning achievement has not been investigated in medical education so far, although both are key factors of PBL success. Thus, we intended to analyse videos of digital problem-based learning (dPBL) sessions, focusing on knowledge acquisition and interaction dynamics among groups with different levels of prior knowledge to reveal any distinctions.

**Methods:**

This study employed a pilot design by dividing 60 dental students into twelve subgroups with less or more prior knowledge, determined by a pre-semester multiple choice test (MCQ). The groups engaged in videotaped dPBL cases, which were examined regarding group interactions and tutor effectiveness. The learning achievement was assessed through a post-semester MCQ, an oral and practical exam.

**Results:**

The video analysis showed that dPBL groups with less prior knowledge achieved significantly higher tutor effectiveness and group interaction utterances, but that the percentage of time in which utterances occurred was similar in both groups. Related to the MCQ results, the students with less prior knowledge learned four times more than those with profound previous abilities, but no significant difference was found in the results of the oral exam and practical exam.

**Conclusions:**

The interaction dynamics in dPBL depend on the group’s amount of prior knowledge. Especially groups including participants with less prior knowledge seemed to benefit from dPBL in comparison to groups with more prior knowledge. The dPBL groups acquired knowledge in different ways during the courses but, finally, all students arrived at a similar level of knowledge.

## Zusammenfassung

**Zielsetzung:**

Forschungsbefunde zum problemorientierten Lernen (POL) zeigen, dass Untersuchungen von Videoaufzeichnungen tutorieller Lernsitzungen bedeutsame Einblicke in kognitive Prozesse ermöglichen. Der Einfluss von Vorwissen auf Lernerfolg unter Einbezug der Lehr-Lern-Interaktionsdynamik wurde in der medizinischen Ausbildung bisher allerdings noch nicht untersucht, obwohl die Faktoren eine Schlüsselrolle für den Erfolg von POL darstellen. Ziel der Studie ist es daher, digitale problemorientierten Lernsitzungen (dPOL) anhand von Videoaufzeichnungen zu analysieren und dabei Wissenserwerbsprozesse und die Interaktionsdynamik in den Lerngruppen in Abhängigkeit vom Vorwissensstand zu untersuchen.

**Methoden:**

In dieser Studie wurde ein Pilotdesign angewandt, bei dem 60 Zahnmedizinstudierende in zwölf Untergruppen mit geringerem oder höherem Vorwissen eingeteilt wurden. Die Erhebung des Vorwissens erfolgte durch einen Multiple-Choice-Test (MCQ) zu Beginn des Semesters. Die Gruppen bearbeiteten mit tutorieller Unterstützung dPOL-Fälle. Die Gruppeninteraktion und Tutor*innenaktivitäten wurden videographiert. Der Lernerfolg wurde am Ende des Semesters anhand eines MCQ sowie einer mündlichen und praktischen Prüfung erhoben.

**Ergebnisse:**

dPOL-Gruppen mit geringerem Vorwissen weisen eine signifikant höhere Anzahl an Aussagen in Gruppeninteraktionen und eine höhere Tutor*inneneffektivität auf. Der zeitliche Umfang der Äußerungen (Prozentsatz, die Äußerungen an der Gesamtzeit einnehmen) ist jedoch in beiden Gruppen gleich. Studierende mit geringerem Vorwissen zeigen einen höheren Lernzuwachs im MCQ. Signifikante Gruppenunterschiede in den Ergebnissen der mündlichen und praktischen Prüfung zeigen sich allerdings nicht.

**Schlussfolgerungen:**

Unterschiedliches Vorwissen der Lernenden führt zu unterschiedlichen Interaktionsdynamiken im dPOL. Im Hinblick auf Lernoutcomes profitieren insbesondere Lernende mit geringerem Vorwissen von dPOL. Die jeweiligen dPOL-Gruppen erreichten am Ende des Semesters ähnliche Lernergebnisse, die Befunde legen aber nahe, dass sich die Charakteristik des Wissenserwerbprozesses in Abhängigkeit vom Vorwissen unterscheidet.

## Introduction

More than 50 years after its first implementation, problem-based learning (PBL) continues to be approved and has spread throughout medical education, enhancing students’ abilities and preparedness for real-life challenges [1]. 

Research in the last decade has revealed several benefits of PBL as being a more enjoyable experience that can foster students’ knowledge construction processes and it has been described that participants relish the self-directed nature of PBL [2]. Students who frequently take part in PBL sessions achieve notably better results in assessments of knowledge and evaluate it as a more pleasing way to study than teacher-centred lectures [3]. Furthermore, current developments such as the interprofessional PBL also showed a high level of acceptance among the participants [4].

There are even educators who consider PBL as a universal remedy for educational shortcomings while others vehemently refuse its implementation and call the impact and value of PBL on learning into question due to its unconventional philosophy and instructional practices [5], [6]. Indeed, there is also evidence that traditional courses tend to lead to an increased achievement of scientific knowledge in comparison to PBL [7]. 

Today we know several factors affecting the outcomes of learning in PBL: the way it is used in various domains, for participants of different ages and subject matters are some of the parameters that influence PBL results [8]. The effectiveness of PBL and the participants’ learning are largely dependent on the tutors’ pedagogical abilities, academic background, training concept and maintenance to the quality of discussion [9], [10], [11], [12]. However, there is still no final conclusion on the best way to optimise PBL-teaching [13].

During the COVID-19 pandemic, PBL research showed new insights regarding synchronous online learning and many faculties provide meaningful results by digitalising their curricula into an e-learning platform [14], [15], [16], [17], [18], [19], [20], [21], [22]. Furthermore, it has been described that direct or videotaped observations or corpus analysis of recordings develop unadulterated information about cognitive processes in small groups [23], [24]. Previous research on the analysis of group interactions and tutor effectiveness in PBL has shown promising results [24], [25], [26], [27], [28], [29], [30], [31], however, the influence of the amount of prior knowledge on learning achievement has never been investigated in medical education, although both are considered as key factors of PBL success [32]. Research in other sciences, e.g. social science and mathematics, has already successfully investigated this relationship and clarified its significance [33], [34].

Due to the current state of research, we intended to analyse videos of digital PBL (dPBL) sessions to concentrate on group interactions, tutor effectiveness and knowledge acquisition between groups with a different amount of prior knowledge. To search for distinctions of both groups and to receive multi-perspective options, videotaped dPBL sessions, multiple-choice tests (MCQ), a structured oral exam (SOE) and an objective structured practical exam (OSPE) were analysed.

Research questions:

- Are there significant differences regarding the group interactions between the two groups?
- Are there significant differences regarding the tutor effectiveness between the two groups?
- Are there significant differences regarding the knowledge acquisition of the two groups?

## Materials and method

### 1. Context and participants

This study was conducted at the dental school of the Goethe University in Frankfurt, Germany. 

The students of the first clinical semester (n=60) were divided into two groups assessed by their prior knowledge, which was amongst others determined by a completion of a pre-semester multiple-choice test (MCQ). The division of the two groups was based on the number of points reached in the pre-semester multiple-choice test, the preclinical examination grade and the general qualification grade for university entrance in Germany (Abitur). The gender also played a role in the division so that women (n=37) and men (n=23) were equally distributed in the final twelve dPBL subgroups. Students with less prior knowledge were assigned to group A (n=30) and students with more prior knowledge were assigned to group B respectively (n=30). Both groups were further separated into subgroups (A 1-6 and B 1-6) using the above described parameters to establish similar subgroups, each with five participants and a tutor. 

During the semester, the twelve groups worked on five dPBL cases. Based on the seven-step PBL model established at the University of Maastricht [35], the first five steps were processed in the first digital session, the sixth step in a self-study period and the seventh step in the second digital session. As Barrows [36] recommended, an eighth step was added in the second digital session to evaluate the dPBL process.

An established expert (Master of Medical Education) trained the tutors on how to supply dPBL in a so-called non-facilitative and facilitative style [37]. Tutors were instructed first to guide the dPBL sessions in a facilitative style, then, as the students gained more experience throughout their dPBL cases, the tutors were told to guide them in a non-facilitative manner. All of the tutors were dentists and each of them attended to a group of students with less prior knowledge and a group of students with more prior knowledge. 

Neither the students nor the tutors knew about the assessment of previous knowledge and the determination to group A or B (double-blind setting). Finally, the sessions were accomplished on Vydeo^®^ (Vydeo, Berlin, Germany), an online platform where the students and the tutor could see each other via video camera and were able to work on a shared document. At the end of the semester, the students were required to pass a post-semester MCQ, a graded SOE and OSPE.

### 2. Video material and coding scheme

#### 2.1. Setting

For the quantitative and qualitative analysis of the videotaped dPBL sessions the software Interact^®^ (Version 18, Mangold International GmbH, Arnstorf, Germany) was used. A second rater independently coded seven hours of a randomly selected part (about 20% of the total video material [26]) after passing a two-week rater training. The inter-rater reliability to Cohen’s kappa was 0.81, thus the first rater analysed all subsequent videos.

#### 2.2. Analysis variables and coding scheme

Based on Visschers-Pleijers’ coding scheme [26], we analysed three different types of group interaction: learning-orientated interactions (i.e. exploratory questioning, cumulative reasoning, and handling conflicts about knowledge), procedural interactions and irrelevant/off-task interactions (see table 1 (Tab. 1)). Irrelevant/off-task interactions contained the subtype “a period silence” which was also measured and analysed.

Furthermore, we examined the tutor effectiveness using a coding scheme based on the evaluation sheet developed by Dolmans [38] (see table 2 (Tab. 2)) as video analysis criteria. Therefore, four approaches of how the tutors stimulated the students’ learning were observed: constructive/active learning, self-directed learning, contextual learning and collaborative learning. The intra-personal behaviour as a tutor was also examined.

**Figure 1:**
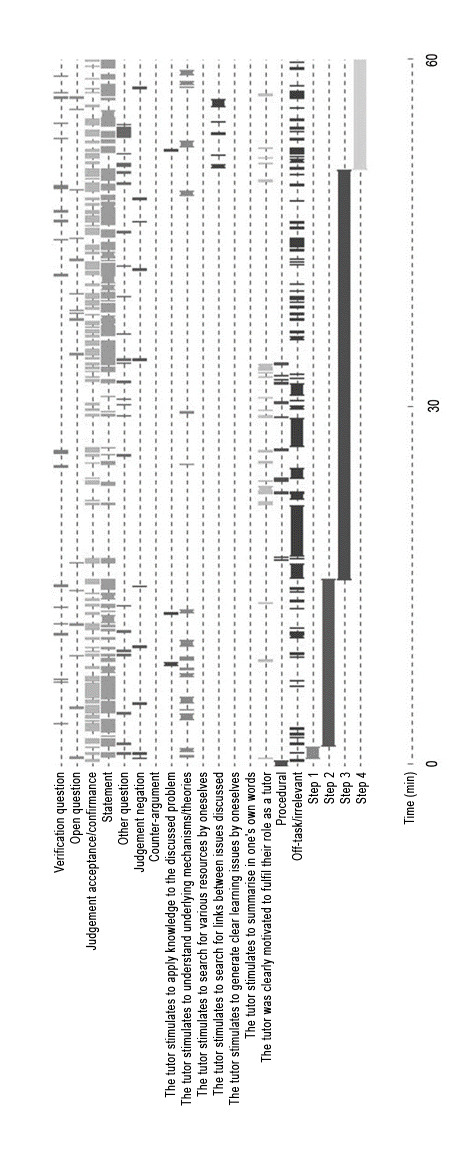
A typical coding result of the first 60 minutes of a dPBL session including steps 1-4. The codes “statements” and “judgement confirmation” are predominant, while at the beginning of step 3 mainly “off-task/irrelevant” codes occurred.

As soon as a participant (student or tutor) made an utterance, with a possible range from one word to several sentences, it was classified according to the given criteria [26], [38]. Every single utterance of the students and the tutors of both groups was examined by the frequency of occurrence and as a percentage of the total session time. From the moment that the utterance began, it was coded and its length determined (see figure 1 (Fig. 1)). Thus, a total of 12,766 student utterances and 1,476 tutor utterances were examined.

### 3. Multiple-choice test

The pre- and post-semester MCQ contained 50 questions that dealt with dental materials and instruments, dental treatment instructions and diagnoses. The MCQ content was also included in the dPBL sessions. The tests were based on a validated template, a previous analysis of which showed a Cronbach’s α of 0.63 (pre-test) and 0.67 (post-test) [37].

### 4. Statistical analysis

In order to analyse the pre- and post-semester MCQ, SOE and OSPE data, the software BiAs^®^ (Version 11.12, Frankfurt, Germany) was used and significant differences (p<0.05) were identified by using a Wilcoxon-Mann-Whitney U Test. 

The video rater’s data were examined using mixed effect models as linear mixed models and generalised linear mixed models [39]. An Interact^®^ software tool especially developed for analysing inter-rater reliability was used to check Cohen’s kappa.

A rough sample estimation was adjusted by examining several factors. Firstly, the PBL time analysed in previous research or the number of PBL sessions conducted were examined as reference points [25], [26], [27], [29], [40], [41], [42], [43], [44], [45], [46]. Secondly, the units of analysis, such as individual videos or segments within videos, were evaluated. By averaging these aspects, we found that at least 15 videos were needed to gather statistically significant information for this pilot study. 

### 5. Application for ethical approval

According to the chairman of the ethics committee of the Goethe University in Frankfurt, no ethical approval was required, because relating to the video material, being recorded was voluntary. The participants knew that they were recorded on video and could approve or decline the recording before each session was started. There were no disadvantages for the participants if they did not agree to be videotaped. All data protection requirements were met. Participation in the dPBL sessions and all exams was mandatory to pass the semester.

## Results

### 1. Analysis of the video data

As the recording of the dPBL sessions was optional, 34 out of 120 PBL sessions were recorded and analysed (dropout rate of 70.9%). The 34 videos consisted of 15 videos from group A with less prior knowledge and 19 videos from group B with more prior knowledge. In both groups the first and second dPBL sessions were evenly divided.

#### 1.1. Learning-orientated interactions analysis

Both groups spent most of the time on cumulative reasoning (group A: 67.3%; group B: 65.7%), while procedural tasks took the least time in both groups (group A: 2.5%; group B: 1.9%). The data group with the highest score of utterances was cumulative reasoning (group A: n=364.9; group B: n=229.6), while the most rare utterances concerned procedural (group A: n=18.7; group B: n=5.1).

Related to the frequency of the occurrence of group interactions, group A scored significantly more utterances in handling conflicts about knowledge, open questions, other questions, statements, disagreements and in procedural interactions (all p<0.05), than group B. There was no significant difference between the groups in the percentage of total session time spent on that types of interactions (see table 1 (Tab. 1)). 

#### 1.2. Tutor effectiveness analysis

Most utterances in the tutor data groups were focussed on constructive/active learning in both groups (group A: 59.3%; group B: 58.3%). The intra-personal behaviour of the tutor engaged the least amount of time (group A: 5%; group B: 7.6%). Constructive/active learning was the data group with the most tutor utterances (group A: n=39; group B: n=30.2), while the least time was spent in collaborative learning (group A: n=4.3; group B: n=2.5) (see table 2 (Tab. 2)).

### 2. Knowledge acquisition 

Group A scored significantly fewer points in the MCQ pre-test but had significantly more points in the MCQ post-test, both compared to group B (see table 3 (Tab. 3)).

Group A had a significant increase of knowledge of 13.7 points and group B of 3.7 points. On average, all students significantly increase their knowledge of 8.7 points (see table 4 (Tab. 4)).

There were no significant differences between both groups in structured oral exam or objective structured practical exam grades (see table 5 (Tab. 5)).

## Discussion

This study’s scientific relevance lies in exploring significant differences in group interactions, tutor effectiveness, and knowledge acquisition between two groups having a different level of prior knowledge. By investigating these aspects, it contributes to understanding the dynamics of group interactions, the effectiveness of tutors, and the impact of prior knowledge on knowledge acquisition. These findings provide valuable insights for educational practitioners and researchers, informing the design and implementation of instructional strategies and interventions to enhance group interactions, optimize tutor effectiveness, and promote effective knowledge acquisition in educational settings. Furthermore, this research provides new information into the influence of prior knowledge to dPBL functioning and gives an example of how dPBL could still be used and analysed during pandemic situations.

Although this study is titled as a pilot study, we examined almost 34 hours and 26 minutes of video material including 14,366 utterances; this is substantially more material than comparable studies based on analysing group interactions or tutor effectiveness in PBL [24], [25], [26], [27], [28], [29], [30], [31], [40], [41], [42], [43], [44], [45], [46], [47], [48], [49], [50], [51], [52], [53]. Nevertheless, we have scheduled a follow-up study with an exact sample estimation to verify our findings and to produce further results.

### 1. Group interaction 

Students with less prior knowledge (group A) performed significantly more utterances on several types of interactions with nearly the same time spent on that interactions as those with more prior knowledge (group B), because group A’s utterances were mostly shorter. 

A possible explanation is that group may not have had the prior knowledge to discuss, argue, ask or explain in depth with each other due to the missing prior knowledge [54], [55]. Additionally, it is also possible that a PBL task is more cognitively activating for one group of learners compared to another, depending on the groups level of prior knowledge. The concept of cognitive activation stems from constructivist learning, refers to the adaptivity of different problems and tasks for different learners and involves evoking cognitive conflicts to initiate learning processes [56], [57]. It might be beneficial to include the measurement of how learners were cognitively activated by the tasks in subsequent research. Cognitive activation can be assumed to be affective for subsequent group discussions, whereby groups with less activating tasks should be less engaged in the discussion. 

Therefore, group A showed a higher rate of conversation tasks and they exchanged information more often which may have supported the elaboration of their prior knowledge [49].

Furthermore, we cannot confirm the results of Visschers-Pleijers et al. [26] and De Grave et al. [58] who believed that if pre-set working rules and role division are retained, then off-task interactions would rarely be measured. Our observation suggests that a period of silence in dPBL was mostly used by students as an “individual thinking period” before the group consensus was reached, an idea that Gukas et al. [47] also considered. In further research, it would be useful to create “silence” as a new topic of interaction, without it being a part of the off-task interactions, because there is evidence that the groups scored highly on the indices of learning, even when they kept silent for some time and that the periods of silence did not indicate that the students were not learning effectively [44], [47].

### 2. Tutor effectiveness 

The only difference concerning the tutor effectiveness was that in group A, the tutor stimulated the students significantly more often to understand the underlying mechanisms/theories regarding the frequency of occurrence. In relation to the percentage of the total session time, there was no difference between the groups on this point. These findings show that the tutors spent less time per utterance for stimulating students with less prior knowledge to understand the underlying mechanisms/theories. A possible reason for this is that the students with more prior knowledge may have demanded explanations of the underlying mechanisms in more detail while group A may have been satisfied with less profound statements.

Overall, the tutors spent more than half of their speaking time on stimulating active learning while only a mere 10%, approximately, on stimulating self-directed learning. Previous research has suggested that self-directed learning is a key to medical skill development in undergraduate curricula [59]. Thus, if the tutors generally spend less time on stimulating self-directed learning, then it may be useful to examine this effect in further research in order to optimise the teaching and training of tutors in the future.

### 3. Knowledge acquisition

In relation to their knowledge acquisition, students with more prior knowledge attained 3.7 points more in the MCQ post-test than in the MCQ pre-test, while students with less prior knowledge achieved 13.7 points more on average. It is remarkable that group A increased their knowledge during the dPBL sessions almost four times more than group B. However, group B started with a relatively high result in the pre-test, so it would be more difficult to raise their own achievement in the post-test (ceiling-effect). In addition, group A scored significantly fewer points in the pre-test and significantly more points in the post-test compared to group B. Some of the tutoring approaches could explain group A’s relatively high level of knowledge acquisition when compared to group B. Firstly, group A had about 180 utterances more per dPBL session on average than group B (group A: 489.3; group B: 306.7); this suggests that there may have been more conversations and more information flow inside group A which would consequently support their knowledge acquisition. Secondly, group A had significantly higher scores in terms of the frequency of occurrence of handling conflicts about knowledge. It is thoroughly valuable to engage on this type of interaction because PBL indicates learning by provoking cognitive conflicts from controversies between the students’ knowledge and the problem they are dealing with [60]. Thirdly, as the participants’ prior knowledge is activated, they become more easily able to find gaps in their knowledge to work on (activation–elaboration hypothesis) and group B may have hindered their knowledge acquisition because they did not have extensive knowledge gaps to elaborate on [61]. Lastly, it could be possible that group A may have prepared themselves better for the dPBL cases, knowing that they had performed worse in the pre-semester MCQ and that they lacked the knowledge of how to deal with the cases instead of trusting in their solid prior knowledge as group B had done. However, there were no significant differences between both groups in the SOE or OSPE grades at the end of the semester. These findings suggest that both groups ended the semester with a similar level of knowledge, but that the two groups acquired this in different ways during the dPBL cases. In addition, dPBL may stimulate the learning process of those participants who had less prior knowledge the most, which may be a possible hint to answer Dolman’s question of “under which conditions is PBL effective and for what kinds of students?” [7]. To answer this question more precisely further research should also consider the ceiling-effect [62].

## Limitations

Several factors limit the generalisability of this study. Firstly, previous research has shown that when being recorded during PBL the participants are not as spontaneous as they are naturally, thus, being videotaped can influence the participant’s behaviour [29]. Furthermore, the participants’ knowledge acquisition may not be entirely based on dPBL as the students also had other lectures and practical trainings during the semester that imparted knowledge. Another point affecting our results is that the participants gained experience during their dPBL cases and this may have changed their group interaction by increasing their expertise or by changing tutoring style (facilitative to non-facilitative [37]). However, our findings only reflect the entirety of the group interaction and, thus, not its trends.

## Conclusion

Interaction dynamics in dPBL depend on the group’s amount of prior knowledge. In particular, groups including participants with less prior knowledge seem to benefit from dPBL by tending towards a fast information flow with shorter utterances in a high frequency in comparison to groups with more prior knowledge. The groups acquired knowledge in different ways during the courses, however, all students did not achieve any significant differences regarding the structured oral exam and objective structured practical exam in relation to their prior knowledge.

## Competing interests

The authors declare that they have no competing interests. 

## Figures and Tables

**Table 1:**
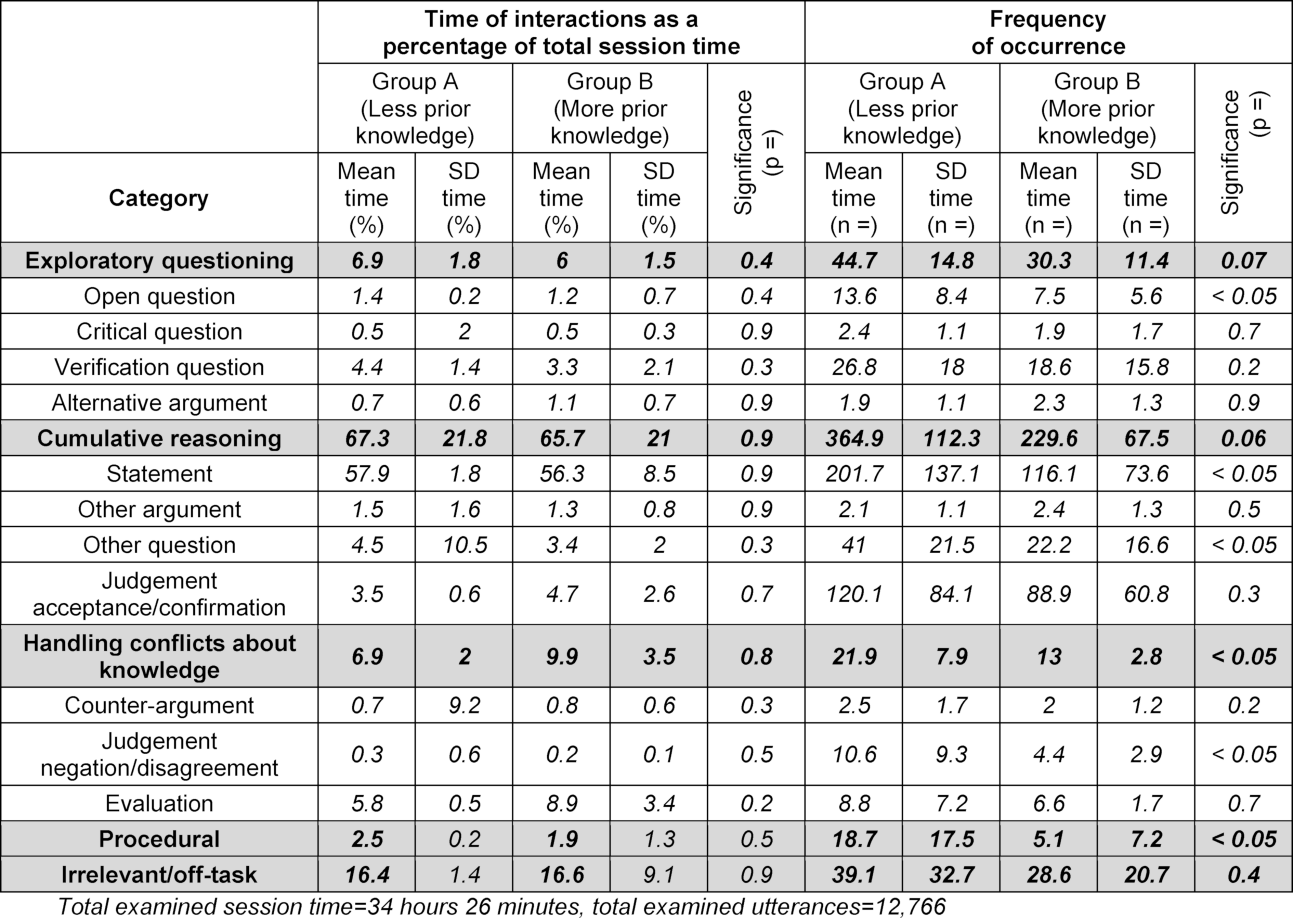
The mean interaction times as percentages of session time and the mean frequencies of the occurrence of utterances in relation to learning-orientated interactions. SD=standard deviation.

**Table 2:**
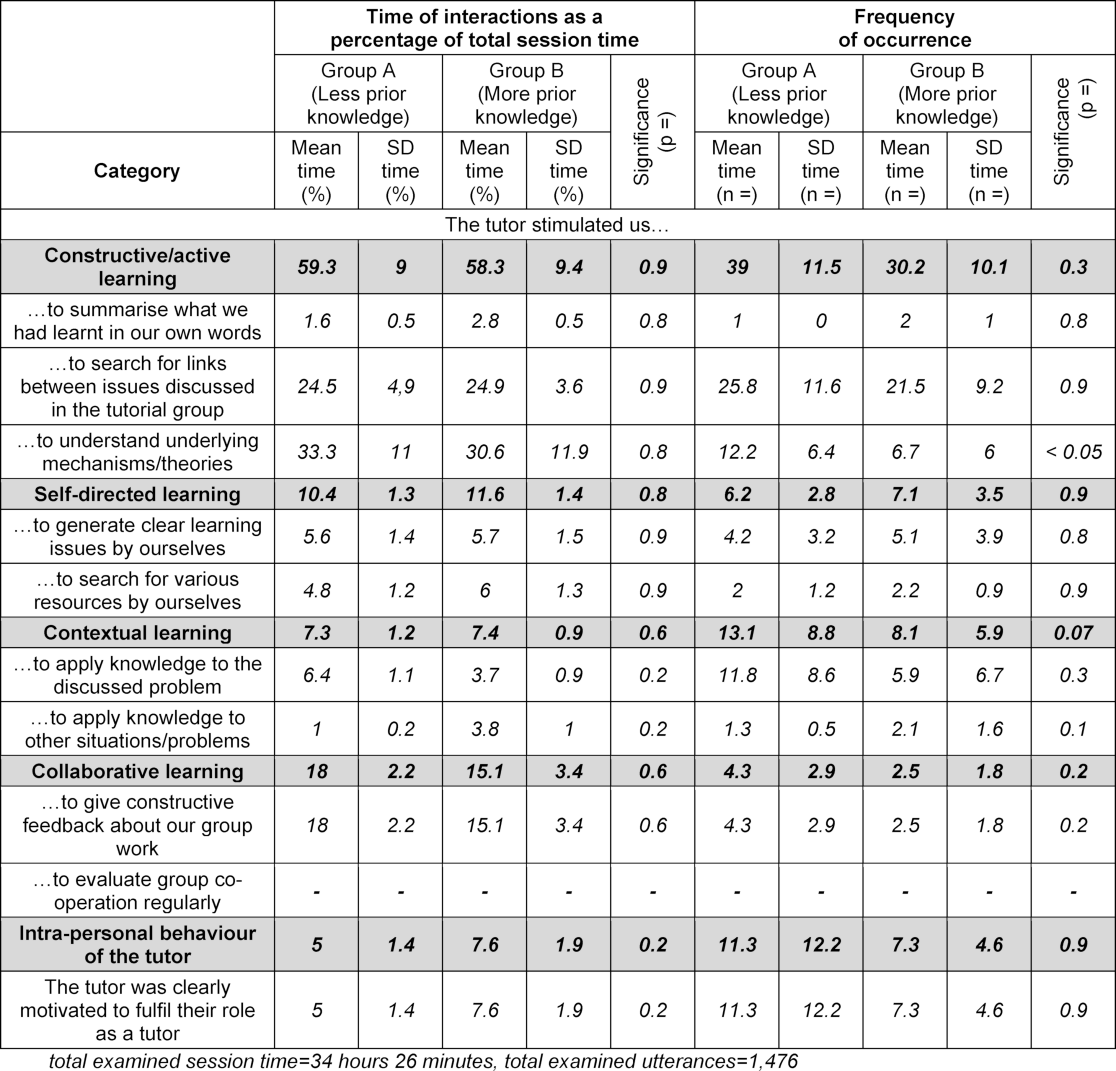
The mean percentages of the session time related to tutor utterances and the mean frequencies of tutor utterances. SD=standard deviation

**Table 3:**
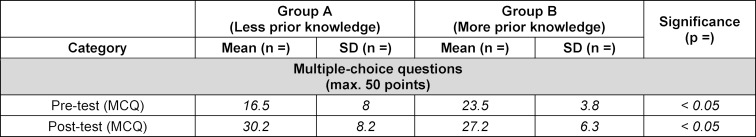
The multiple-choice questions (MCQ) grades in relation to group A and B. SD=standard deviation

**Table 4:**
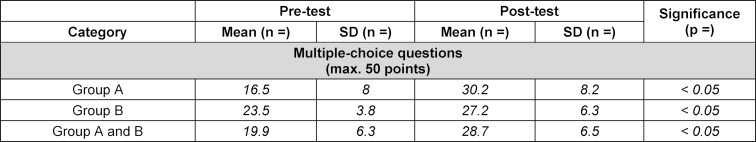
The multiple-choice questions (MCQ) grades in relation to the pre- and post-test. SD=standard deviation

**Table 5:**
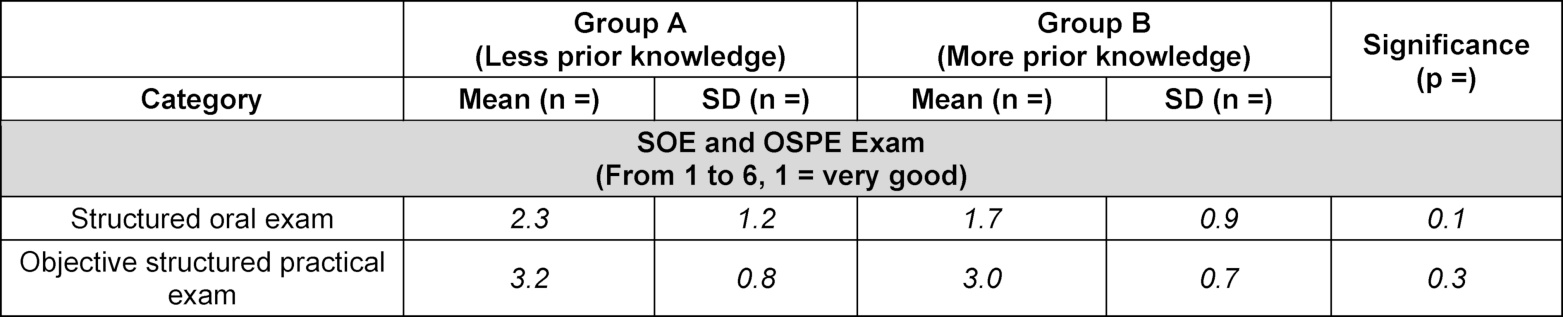
The objective structured practical exam (OSPE) and structured oral exam (SOE) grades. SD=standard deviation
